# Flow of Medication Information Incidents in the Home Care Setting in Finland: A Qualitative Descriptive Study

**DOI:** 10.1111/jan.70063

**Published:** 2025-07-04

**Authors:** Marja Vellonen, Marja Härkänen, Tarja Välimäki

**Affiliations:** ^1^ Department of Nursing Science University of Eastern Finland Kuopio Finland; ^2^ Laurea University of Applied Sciences Vantaa Finland; ^3^ Research Centre for Nursing Science and Social and Health Management Kuopio University Hospital, Wellbeing Services County of North Savo Kuopio Finland

**Keywords:** healthcare professionals, home care, information flow, interview study, medication incident, nursing, qualitative study

## Abstract

**Aim:**

To describe the challenges related to the flow of medication information in home care, their contributing factors, and home care registered nurses' and nurse leaders' views on preventing them.

**Design:**

A descriptive qualitative study.

**Methods:**

Six group and one individual semi‐structured interview were conducted remotely with 15 home care registered nurses and nurse leaders between 12 February 2023 and 9 November 2023 in Finland. The data were analysed using reflexive thematic analysis.

**Results:**

We identified four main themes related to the challenges of medication information flow: the complexity of home care work in cooperation and the medication process, technology‐related challenges, the healthcare professionals' individual factors and client‐related challenges. These factors contributed to the challenges: the lack of healthcare professionals' resources, the healthcare professionals' attitudes to work and individual characteristics, the lack of healthcare professionals' uniform practices and client‐related factors. Preventing challenges and incidents: strengthening standard healthcare practices, increasing healthcare resources, improving the individual factors of healthcare professionals, and guiding the client in the management of medication.

**Conclusion:**

The medication information flow can be improved by discussing standard practices for the flow of medication information in home care and between home care and hospital teams.

**Implications for the Profession and/or Patient Care:**

It is crucial to identify challenges, contributing factors and prevention in the medication information flow from the home care registered nurses' and nurse leaders' perspective. These elements play an important role in developing medication information flow by collaborating extensively with other healthcare providers, clients, and relatives.

**Impact:**

Healthcare professionals, nurse leaders, and educators can utilise this study's findings to develop the flow of medication information within and between organisations.

**Reporting Method:**

The Standards for Reporting Qualitative Research checklist was used.

**Patient or Public Contribution:**

No patient or public contribution.

**Summary**

- What does this paper contribute to the wider global clinical community?
  ○ Healthcare professionals require standard practices on medication information flow when clients move between various healthcare organisations.
  ○ The number of healthcare professionals in home care should be increased, and special attention must be given to strengthening the language skills of home care nurses to prevent medication‐related incidents.
  ○ Pharmacists, part of the home care team, facilitate the flow of medication information.

## 1 Introduction

Medication incidents in home care are common, causing suffering for clients and their relatives and concern for healthcare professionals (Wu et al. 2020). These medication incidents are often related to communication and information flow challenges (Härkänen et al. 2020; Syyrilä et al. 2020). In this study, medication incidents are defined as events that cause or could cause harm to the client (National Coordinating Council for Medication Error Reporting and Prevention (NCCMERP) 2021).

In this study, home care refers to care provided by healthcare professionals at the client's home and via a remote care device (a tablet computer) (Finnish Institute of Health and Welfare 2023). In Finland, several providers are involved in the medication process, the most important being home care nurses, clients, relatives and pharmacies (Vellonen et al. 2023). When a client moves between different healthcare organisations, responsibility for the client's medication varies significantly. It is crucial to ensure the timeliness and accuracy of information between different healthcare providers to avoid incidents during the medication process. In this study, information flow refers to the flow of information related to all medication documents (e.g., medication records, medication orders) between home care professionals and other providers (e.g., hospitals, relatives, pharmacies).

## 2 Background

Home care is a complex care environment. Its complexity consists of the client's home environment, client group and fragmented healthcare services. Client homes vary and are not designed for professional care (Shahrestanaki et al. 2023), making it challenging for home care professionals to work safely (Geil Kollerup et al. 2018). Moreover, the client's homes have various methods of storing medication. Medications are found in different places, such as on the kitchen table, the top of the mini‐oven, and the bathroom (Geil Kollerup et al. 2018). Additionally, most home care clients are older adults, a vulnerable group for medication‐related incidents due to polypharmacy and reduced functional ability (Squires et al. 2020). However, many older adults live alone (Statistics Finland 2019), and home care professionals spend only limited time at clients' homes (Geil Kollerup et al. 2018). The client may also use healthcare services outside the home, such as public hospital services or private services. In these cases, responsibility for the client's medication is shared among several healthcare providers (Vellonen et al. 2023). Previous studies have shown that fragmented healthcare systems contribute to communication issues between healthcare professionals, leading to an increased risk of medication incidents (Meyer‐Massetti, Hofstetter, et al. 2018; Squires et al. 2020; Vellonen et al. 2023). Communication between various levels of providers is often inadequate, and home care may receive insufficient information (Squires et al. 2020).

Several studies on home care describe communication and information flow (Härkänen et al. 2020; Vellonen et al. 2023) as well as cooperation challenges (Squires et al. 2020; Dionisi et al. 2022; Vellonen et al. 2023) between healthcare professionals as contributing factors to medication incidents, particularly during transitions from hospital to home care (Dionisi et al. 2022; Vellonen et al. 2023). Therefore, this study applied the framework developed by Kuziemsky et al. (2009) for interdisciplinary team communication in palliative care, focusing on the communication structure framework in the home care context in Finland.

Previous studies have observed challenges in the accuracy of discharge documents from hospitals to home care. For instance, Meyer‐Massetti, Hofstetter, et al. (2018) found that only 5% of clients had complete written discharge information. Berland and Bentsen (2017) noted that transition documents were often outdated, or medication information was delayed. Squires et al. (2020) reported that unclear medication instructions left clients uncertain about their medication regimens. Home care professionals describe these situations as challenging, as they spend considerable time clarifying and organising medication information (Geil Kollerup et al. 2018).

Additionally, the e‐message system did not support the information flow between hospitals and home care regarding clients' medication, forcing home care nurses to verify discharge medication accuracy by telephone (Foged et al. 2018). Vellonen et al. (2023) found that different patient record systems between hospitals and home care led to delays in information flow. Studies have shown that pharmacists can help prevent medication‐related incidents (Dionisi et al. 2021), especially during the transition of care to other healthcare providers (Kee et al. 2018; Meyer‐Massetti, Meier, and Guglielmo 2018).

In home care, medication incidents related to information flow can also occur between home care professionals. Vellonen et al. (2023) reported finding outdated and incorrect medication documents in home care clients' homes, contributing to information flow incidents. They observed that the lack of information‐sharing, slowness and informal communication between healthcare professionals lead to medication incidents. Berland and Bentsen (2017) highlighted communication challenges between physicians and home care nurses, with physicians failing to provide necessary medication information in a timely manner. The global shortage of nurses, which is noticeable in Finland (Cubelo et al. 2023), further complicates the situation. The shortage has led to the use of qualified substitute nurses in home care organisations because there are not enough permanent home care nurses. This presents challenges for information flow from substitute nurses to permanent staff (Vellonen et al. 2023).

Various factors contribute to this complexity, including a lack of resources (Härkänen et al. 2020) and inadequate competence (Berland and Bentsen 2017), both of which increase the likelihood of medication incidents. Home care nurses believed that medication incidents were partly due to hospital nurses' heavy workloads and increasing responsibilities (Foged et al. 2018). Berland and Bentsen (2017) also found that home care workers lacked adequate training in medication management. Moreover, language skill challenges were identified (Vellonen et al. 2023). For example, Vellonen et al. (2023) found that some nurses had poor language skills. Therefore, they failed to write entries in the patient record system. One common challenge was the lack of a shared language between home care nurses and their clients. Squires et al. (2020) observed that some clients lacked language skills and could not read or understand their medication instructions. They also identified other client‐related factors for medication incidents, such as clients using multiple healthcare services and pharmacies instead of one. Simultaneously, a home care client's relative may participate in the client's medication management and take the client to another healthcare setting where medication changes are made (Vellonen et al. 2023). In such cases, it is especially important to guide clients on their medication regimen (Härkänen et al. 2020).

Globally, efforts have been made to improve patient safety. The Global Patient Safety Action Plan 2021–2030 provides a strategic direction for stakeholders to minimise harm and improve patient safety (World Health Organization 2021). The implementation of safe medication practices is supported by checklists, such as ensuring that the right client receives the right medication, in the correct dose, at the correct time, and by the correct route. However, checklists alone are insufficient. Therefore, it is essential for healthcare professionals and organisational leaders to discuss safe practices (Institute for Safe Medication Practices (ISMP) 2023). Research on client safety has recently become a focus in various areas of healthcare, including home care, but data on medication incidents in home care remain limited (Shahrestanaki et al. 2023). Notably, findings on the flow of medication information incidents between healthcare professionals, clients, and relatives are still lacking.

### 2.1 Home Care in Finland

By 2022, there were approximately 194,000 home care clients in Finland, most of whom were older adults. Regular home care services were used by 59% of clients (*n* = 114,091) (Finnish Institute of Health and Welfare 2022). In Finland, the public sector provides home care services in clients' homes or remotely via tablet computers, available around the clock. The private sector complements these services. Various types of care can be provided at home, such as supporting clients in their daily activities and managing medication. Remote technology can remind clients to take their medication via phone or video connection (tablet computer) (Finnish Institute of Health and Welfare 2023). The client's medications can be dispensed into a dose box by home care professionals or at a pharmacy, where the client receives dose delivery bags at home. Additionally, clients may use a medicine‐dispensing robot at home.

In 2021, 17,000 personnel worked in home care in Finland. Of these, 74% were practical nurses (with an upper secondary level qualification from a vocational school, 180 ECTS credits), and 12% were registered nurses (with higher education from a University of Applied Sciences, 210–270 ECTS credits), both referred to in this study as home care professionals (Finnish Institute of Health and Welfare 2023). All nurses had formal licensed or registered education. Registered nurses are responsible for managing clients' overall medication care at home, including administering intravenous medications. Practical nurses administer and dispense medications. Both nursing groups require medication licences, which are controlled at the organisational level by nurse leaders or directors. Employers must ensure that their staff have sufficient competency and language skills to carry out medication care. A physician (with higher education from a university, Licentiate of Medicine, 360 ECTS credits) is responsible for overseeing the client's medication, particularly in prescribing medications. In Finland, either the home care physician or health centre physician is responsible for the home care client's medication in primary care.

Unit‐specific medication plans ensure safe medication care in Finland's healthcare organisations (Valvira 2024). Healthcare organisations report medication‐related incidents via the incident‐reporting system.

## 3 The Study

### 3.1 Aim

To describe the challenges related to the flow of medication information in home care, their contributing factors, and home care registered nurses' and nurse leaders' views on preventing them.

The research questions were:

- What are the challenges related to the medication information flow?
- What factors contribute to the challenges in the medication information flow?
- How can challenges and incidents related to the medication information flow be prevented?

## 4 Methods

### 4.1 Design

This study used a qualitative descriptive approach, using group and individual semi‐structured interviews. A qualitative descriptive approach aligns with a constructivist paradigm, wherein reality is multiple and grounded in the participants' subjective experiences (Polit and Beck 2021). This approach allowed registered nurses and nurse leaders to comprehensively describe their experiences in their own voice, which is particularly important for advancing home care practices (Doyle et al. 2020).

### 4.2 Theoretical Framework

This study utilised the framework developed by Kuziemsky et al. (2009), focusing on interdisciplinary team communication in palliative care. Their framework includes team meta‐concepts of structures, processes and outcomes. This study uses the communication structure framework to describe the flow of medication information among various healthcare professionals, clients and relatives in home care. Kuziemsky et al. (2009) described structures from internal and external teams' perspectives. The internal team perspective focuses on how effectively the team functions, including membership, policies, procedures and communication practices. The external team perspective considers how teams coordinate with outside agencies and/or individuals, using communication channels such as telephone and email.

### 4.3 Study Setting and Recruitment

This study was conducted in a home care setting in one city in Finland. All registered nurses and nurse leaders in home care were invited to participate in the study through their leaders using snowball sampling. Home care nurse leaders were the registered nurses' administrative managers. The nurse leaders distributed the study invitation and consent forms to the home care registered nurses in their work organisations. Subsequently, the volunteer study participants contacted the first author of this paper by email to arrange an interview time. Before the interview, the participants received the interview themes via email from the first author (Table 1). In addition, participants' demographic and professional information was collected.

**TABLE 1:** Group and individual semi‐structured interview guides (Brinkmann and Kvale 2018; Vellonen et al. 2023).

**Beginning of the interview** *Warm‐up questions* How are you? How has your day been? Participant's introduction (in the individual interview) Participants are introduced to each other (in the group interview) **General information** The interviewer gives information about the study and the opportunity to ask questions. Defining what medication information flow means in this study. **Interview themes** *Theme I*: The challenges related to the flow of medication information in home care and the factors that contribute to them at the different stages of the medication process. Questions: What are the challenges related to the medication information flow? What factors contribute to the challenges in the medication information flow? *Theme II*: Prevention of home care medication challenges and incidents related to the information flow at the different stages of the medication process. Question: How can challenges and incidents related to the medication information flow be prevented? The interviewer used the following question formats in the group and individual interviews to keep the conversation going and clarify the participants' expressions. *Follow‐up questions* Can you tell us more about this challenge and/or its contributing factors? What kind of situations are these challenges related to? Can you elaborate on how to prevent this challenge or incident? *Probing questions* Could you say something more about that…? Can you give a more detailed description of what happened…? Do you have further examples of this…? *Interpreting questions* You then mean that …? Is it correct that…? **At the end of the interview** Do you want to tell us more about the themes? Do you have any questions about the interview? Thank you for participating

### 4.4 Inclusion Criteria

The inclusion criteria for participating in the study included registered nurses and nurse leaders currently employed in home care in one city in Finland.

### 4.5 Data Collection

Between 12 February 2023 and 9 November 2023, we gathered data from group and individual semi‐structured interviews with home care registered nurses and nurse leaders. The first author conducted the interviews remotely (Microsoft Teams Meetings) at a time convenient for the participants. The interview guide directed the flow of the group and individual semi‐structured interviews (Brinkmann and Kvale 2018), which were pre‐tested with one group before the actual interviews. The research questions and previous studies on the flow of medication information incidents guided the theoretical development of the interview guide (Vellonen et al. 2023). Follow‐up, probing and interpretative questions were used to clarify the participants' expressions (Brinkmann and Kvale 2018) (Table 1). The group interviews were mainly conducted in groups of two to four participants (*n* = 14), comprising six groups. Some participants were familiar with each other in the interview. The interviewer moderated the discussions to ensure the groups stayed on topic and that all participants were heard. One participant (*n* = 1) was interviewed individually.

Based on the Kuziemsky et al. (2009) framework, participants were informed at the beginning of the interviews about the definition of medication information flow in this study. Medication information flow was understood as a broad entity, including the concept of communication. Information flows occur between healthcare professionals, home care clients, and relatives about the client's medication information (e.g., medication record, medication orders) at different stages of the medication process. Communication between different healthcare professionals, clients, and relatives can be direct (e.g., face‐to‐face) or indirect (e.g., electronic communication between healthcare professionals via a patient record system). The recorded interviews lasted for 48–69 min, totalling 6 h and 54 min. The first author transcribed the data.

### 4.6 Data Analysis

**FIGURE 1:**
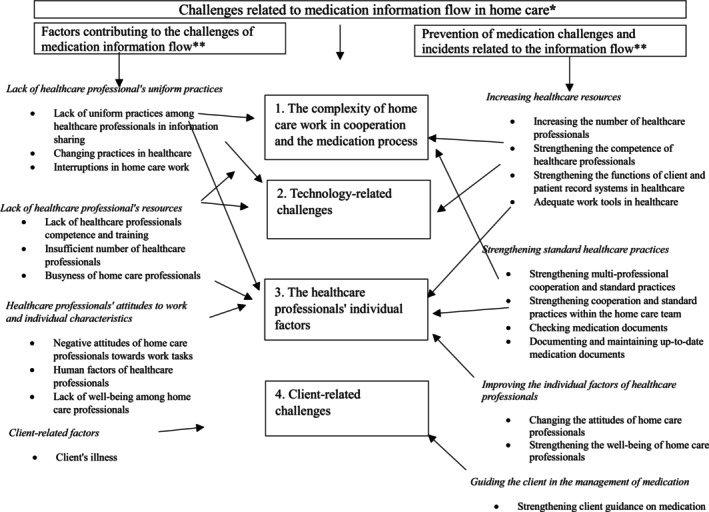
A thematic map: Challenges related to the flow of medication information in home care* and their contributing factors**, and home care registered nurses' and nurse leaders' views on preventing challenges and incidents**. The arrows illustrate the relationships between the themes in the three research questions. *Main themes described in the figure. **Main themes and themes described in the figure.

We conducted reflexive thematic analysis on transcribed data from group and individual semi‐structured interviews, addressing each research question (1–3) separately and inductively (Braun and Clarke 2022). This approach aligns with the constructivist framework, aiming to provide a rich and diverse account of the experiences of registered nurses and nurse leaders (Polit and Beck 2021). The analysis followed these six stages (Braun and Clarke 2022): (1) *Familiarisation with data*: The first author reviewed the transcriptions multiple times to gain an overarching understanding of the challenges in medication information flow (research question 1), contributing factors (research question 2), and strategies for preventing challenges and incidents (research question 3). (2) *Data coding*: The first author coded the data for each research question (1–3) independently. During coding, distinct meanings and concepts were identified and assigned descriptive labels (code labels). (3) Theme generation: Initial themes were created by organising the codes from each research question according to similarities. These were structured into different levels—main themes, themes, and sub‐themes. (4) *Theme development and review*: The first author refined and reworked the themes as needed. A thematic map was then created to visually represent the relationships between themes. Analyses for all three research questions were integrated into this map, which two other researchers subsequently reviewed and provided feedback on. (5) *Finalisation of themes*: With input from the two other researchers, the first author refined, defined, and named the final themes (main themes, themes, sub‐themes) and finalised the thematic map. (6) Report writing: Overall, the analysis of research question 1 contained 100 codes, 22 sub‐themes, 9 themes, and 4 main themes. Research question 2 analysis included 60 codes, 24 sub‐themes, 10 themes, and 4 main themes. Research question 3 analysis consisted of 122 codes, 33 sub‐themes, 11 themes, and 4 main themes. The researchers compiled a report outlining the main themes and themes, illustrated in Tables 3, 4, 5 for each research question and Figure 1, which consolidates all three questions. Direct quotes support the findings.

### 4.7 Ethical Considerations

Our study received a favourable ethical statement on 26 September 2022 from the University of Eastern Finland National Committee of Research Ethics. After that, the study received a research permit on 23 January 2023 for interviews with home care registered nurses and nurse leaders in a city in Finland (Finnish National Board on Research Integrity; TENK 2019).

Before the interview, the participants were informed about the voluntary nature and anonymity of the study, and they provided their consent for participation. Separate interview groups were organised for registered nurses and nurse leaders. Interviews were recorded with the participant's permission and deleted after transcription. If study participation evoked unpleasant memories of unintended medication incidents, the participants were encouraged to discuss these with their nurse leaders.

### 4.8 Rigour and Reflexivity

We assessed the reliability of our study using established qualitative research criteria, including credibility, confirmability, dependability, authenticity, and transferability (Lincoln and Guba 1985; Polit and Beck 2021). Throughout the analysis process, the authors maintained a reflective stance, striving to minimise preconceptions. The analysis was conducted systematically following the principles of reflexive thematic analysis, with all authors reviewing the process and engaging in multiple discussions to refine the findings (Braun and Clarke 2022). These steps reinforced the study's credibility and confirmability (Lincoln and Guba 1985). To ensure dependability and authenticity, the research and analysis procedures were meticulously documented, incorporating direct quotations in the report (Lincoln and Guba 1985; Polit and Beck 2021).

The home care medication process differs between countries. Therefore, the research setting was described in detail to allow readers to assess the transferability of the results to other healthcare contexts (Lincoln and Guba 1985). Additionally, one of the authors had previously worked in home care. Thus, the analysis was performed objectively to avoid subjective experiences. Additionally, we used the Standards for Reporting Qualitative Research (SRQR) checklist to review research reports (O'Brien et al. 2014).

## 5 Findings

### 5.1 Characteristics of Participants

The sample included 12 registered nurses and 3 nurse leaders. The participants had a mean age of 44, with ages ranging from 27 to 62. A majority of the participants were female (87%), while 13% were male. Most participants were registered nurses (80%), and 20% were nurse leaders, all of whom had registered nurse education. Participants' total healthcare experience varied from 4 to 37 years (mean = 18 years), and their home care experience varied from 1 to 27 years (mean = 9 years) (Table 2).

**TABLE 2:** The participants' demographic and professional characteristics (*n* = 15).

Characteristics	Mean (range)
Age (years)	44 (27–62)
Total experience in healthcare (years)	18 (4–37)
Total experience in home care (years)	9 (1–27)
	**N** (%)
Sex	
Female	13 (87)
Male	2 (13)
Work task	
Registered nurse^a^	12 (80)
Nurse leader^a^	3 (20)

^a^ With registered nurse education.

### 5.2 Overview of the Findings

Our analysis identified four main themes related to the challenges of medication information flow in home care: (1) the complexity of home care work in cooperation and the medication process, (2) technology‐related challenges, (3) the healthcare professionals' individual factors and (4) client‐related challenges (Table 3).

**TABLE 3:** Challenges related to medication information flow in home care.

Main theme	Theme
The complexity of home care work in cooperation and the medication process	Cooperation challenges between different healthcare professionals and between healthcare professionals and clients' relatives
	Medication process challenges
Technology‐related challenges	Challenges related to client and patient record systems in healthcare
	Challenges related to well‐being and health technologies and tools in home care
The healthcare professionals' individual factors	Incomplete actions by healthcare professionals
	Incorrect medication information flow between healthcare professionals
Client‐related challenges	The client independently visits healthcare services
	The client does not follow healthcare professionals' instructions regarding medication
	The client's lack of medication information

This report presents the findings from the perspective of the challenges of medication information flow with four main themes, including their individualised contributing factors (Table 4) and home care registered nurses' and nurse leaders' views on preventing medication information flow challenges and incidents (Table 5). The findings illustrated the communication structure framework proposed by Kuziemsky et al. (2009), in which internal and external teams collaborate via communication channels. Teams' awareness of medication information flow policies and procedures was crucial for success. A thematic map illustrates the relationships between themes in the three research questions (Figure 1).

**TABLE 4:** Factors contributing to the challenges of medication information flow in home care.

Main theme	Theme
Lack of healthcare professional's resources	Lack of healthcare professionals competence and training
	Insufficient number of healthcare professionals
	Busyness of home care professionals
Healthcare professionals' attitudes to work and individual characteristics	Negative attitudes of home care professionals towards work tasks
	Human factors of healthcare professionals
	Lack of well‐being among home care professionals
Lack of healthcare professional's uniform practices	Lack of uniform practices among healthcare professionals in information sharing
	Changing practices in healthcare
	Interruptions in home care work
Client‐related factors	Client's illness

**TABLE 5:** Prevention of medication challenges and incidents related to the information flow in home care.

Main theme	Theme
Strengthening standard healthcare practices	Strengthening multi‐professional cooperation and standard practices
	Strengthening cooperation and standard practices within the home care team
	Checking medication documents
	Documenting and maintaining up‐to‐date medication documents
Increasing healthcare resources	Increasing the number of healthcare professionals
	Strengthening the competence of healthcare professionals
	Strengthening the functions of client and patient record systems in healthcare
	Adequate work tools in healthcare
Improving the individual factors of healthcare professionals	Changing the attitudes of home care professionals
	Strengthening the well‐being of home care professionals
Guiding the client in the management of medication	Strengthening client guidance on medication

### 5.3 Main Theme 1: The Complexity of Home Care Work in Cooperation and the Medication Process

Participants described home care work as complex and viewed it as *a challenge*. The key factors contributing to this complexity were cooperation challenges between different healthcare professionals, healthcare professionals and clients' relatives, as well as challenges in the medication process. *The contributing factors* were the lack of uniform practices and resources for healthcare professionals. These *challenges and incidents can be prevented* by strengthening standard healthcare practices and increasing healthcare resources.

Participants mentioned home care client care is typically shared between several healthcare professionals, pharmacies, and clients' relatives. The client's medication information is then divided among several individuals. Consequently, there was a risk that healthcare professionals or relatives who were caring for the client at the time did not have access to the client's updated medication information. In addition, participants indicated that communication between healthcare professionals might be delayed in the transfer of medication information, leading to a medication‐related incident. This complexity increased when the client's medication was changed multiple times within a short period. Contributing factors to this included busyness, insufficient healthcare professionals, and a lack of competence.

…there are many people, and many different factors involved [in the medication process]; there is a physician; a registered nurse; a practical nurse; a pharmacy/pharmacist. Someone goes to get the medication, someone distributes the medication, and someone delivers the medication to the client. There are so many, many different stages. If you think about the information flow…. (*challenges*)—Interview 2

…there may be a delay, for example, the paper medication list coming home with the discharge papers in an envelope to the client, and information that there is such a list with changes [in the client's home] might come to home care with a delay. For example, in the worst case, the client has received a few days' worth of medications from the discharging organisation, and the home medication continues according to the old client information in the home care patient record system, as [home care] was not aware that there was a need for updating. (*challenges*)—Interview 7

Home care professionals used diverse communication tools, including phone and electronic communication via the patient record system. The client's medication information was not always transferred to all home care team members, which participants described as ‘silent reporting’. Silent reporting meant that a client's medication was verbally communicated to a colleague but not documented in the patient record system. Communication could occur informally, such as through Post‐it notes. In such cases, participants mentioned there was a risk that the client's medication information would not be passed on to another healthcare professional. This kind of delegation was unnecessary; participants encouraged the home care professionals to complete their work as much as possible, which would prevent medication information flow challenges and incidents.

…there [in the home care office] is a Post‐it note; there was some medication end [at the client's home]…you [nurse] leave the Post‐it note [for another nurses], and then they are floating on the floor [of the home care office]. Some of these really critical things like medication; it would be nice if they were carried through to the end. (*challenges and contributing factors*)—Interview 6

Complete tasks to the end [avoiding unnecessary delegation about the medication], especially when the client is being discharged. (*prevention*)—Interview 6

However, participants described that home care often did not receive information about a client's medication changes from the hospital, health centre, private health service, remote care, or the client's relatives. Participants identified the need for joint discussions between home care and hospital teams on how to manage the information flow regarding medication, especially when the client is returning home. A hospital nurse familiar with the client should call home care upon the client's transfer. Moreover, it was important to agree on standard practices for medication management between home care professionals, hospital professionals, and the client's relatives to prevent medication information flow challenges and incidents.

…if the physician only records information in the patient record system on the narrative page, and not necessarily on the medication list. This medication thing does not get forwarded, and we are not informed. We only find out later that there was a note from the physician's in the text. Consequently, there are problems with information flow. (*challenges and contributing factors*)—Interview 3

Yes, there should be more cooperation [with the hospital], especially with specialised medical care physicians, cooperation meetings, and clear rules, guidelines on how to do proceed [the information flow], and it should be same for everybody. (*prevention*)—Interview 3

Participants described that home care and pharmacies need standard practices for information flow and communication, which help prevent medication information flow challenges and incidents. Participants further described that working with a familiar pharmacy contributed to medication safety, as the pharmacy was familiar with the client's medication and stayed in touch with home care when they noticed anything unusual. Participants also felt that medication information flow improved when pharmacists worked in home care.

…we have our pharmacist in home care, which increases medication safety and information flow, and everything…[Home care pharmacist] organise this information between home care and the pharmacy and keep us well informed. (*prevention*)—Interview 2

…regarding information flow, our previous pharmacy called very sensitively for us and asked in a friendly manner if you ordered some medication, even if you had just ordered something unusual, they would ask what this is…. (*prevention*)—Interview 2

Participants described that creating uniform practices, using different communication tools could enhance information flow. For example, they mentioned the use of a lean board. The Lean board was displayed on the wall of the home care office. It outlined the key care tasks for clients in the home care area. They also said that consulting colleagues in case of unclear medication issues in the home care team could prevent medication information flow challenges and incidents.

But checklists would surely help [in information flow]; we have lean boards. (*prevention*)—Interview 6

But mainly the foresight work, nurses are often reminded to check at the client's home that there are enough medications for tomorrow and the day after. (*prevention*)—Interview 2

I [a registered nurse] hope that if any medication‐related questions arise for a nurse in the field [client's home], I hope that they will call in case of unclear medication situations…. (*prevention*)—Interview 7

The home care client's medication process involved many steps, from prescribing medication to administering it to the client. A home care client may have medications in a dose box, a dose delivery bag, or a medicine‐dispensing robot. The medication dose delivery bag process was perceived as particularly challenging from an information flow perspective. Participants mentioned that this complexity increased when several healthcare professionals were involved in the medication process, and the client's medications were in multiple locations (e.g., in a dose box and a dose delivery bag).

Medication changes are problematic, especially if the client has medication in the dose delivery bag…if the medication must be started quickly, we have to distribute it separately into the other dose box. (*challenges*)—Interview 3

This is quite common, when there are a medication dose box and a dose delivery bag [at the client's home], especially if there are substitute nurses coming from outside the work community, some pills might be left ungiven more often than by permanent employees. This means that medications are left ungiven from the dose box or the dose delivery bag. If the medications are in two different locations, they must be given at the same time, so there is a risk, and this can cause many incidents. (*challenges*)—Interview 2

The nurses are reminded to check the care plans, medication lists, and dose delivery bags before giving them to the [clients]. (*prevention*)—Interview 2

### 5.4 Main Theme 2: Technology‐Related Challenges

*Challenges* related to home care technology included client and patient record systems, well‐being, and health technologies and tools. The lack of uniform practices and resources among healthcare professionals *contributed to these challenges*. Increased healthcare resources *can prevent challenges and incidents* related to the flow of medication information.

Participants described that the information technology (IT) challenges between home care and pharmacies occurred when the information systems were not working, meaning that information did not flow between providers. The participants said that a contributing factor was a lack of resources for pharmacy staff, particularly in situations where the home care partnership pharmacy changed. In addition, participants described weaknesses in the functionalities of the home care patient record system, which prevented smooth communication between healthcare professionals. This led healthcare professionals to be cautious when a client's medication was changed or a prescription was renewed because the medication administration times disappeared from the daily medication list. In addition, the patient record system informed home care professionals that a client's medication might be out of date 1 day later. The constantly changing characteristics of the patient record system were identified as contributing factors. The participants believed that simplifying the patient record system would prevent medication incidents. However, participants perceived that healthcare professionals competence in using the patient record system was weak and, therefore, needed more training.

Our patient record system does not give any alerts about it, it merely informs you that the medication list might be outdated. But the notification comes after 24 h, and we rarely check the client's medication lists daily. (*challenges*)—Interview 7

The patient record system is constantly changing, it is in a state of continuous change. (*contributing factors*)—Interview 1

…it should perhaps be simplified even more, so that it is not the system's fault [a patient record system]. (*prevention*)—Interview 2

Patient record systems allow healthcare professionals to send messages about their clients. However, participants mentioned that a lack of competency was a contributing factor, meaning the messages did not reach all healthcare professionals through the patient record system. In addition, they said that the work of home care substitutes was made more difficult because they did not have the same work tools as permanent nurses. Home care substitute workers did not always have access to a patient record system that operated on a mobile phone. Consequently, they had to rely on paper documents that could be outdated at clients' homes. Participants wanted the same work tools for home care substitute workers as permanent employees, which were considered to prevent medication information flow challenges and incidents. Moreover, they said that client information was fragmented in the patient record system, making it difficult for healthcare professionals to locate it.

I went to give an injection last week and recorded it in the patient record system… Yes, it's difficult retrieve the patient record system, especially if you don't know how…she/he would have gone to give it again. (*challenges*)—Interview 5

Overall, the lack of a uniform patient record system challenged healthcare professionals, particularly during client transitions from hospital to home care. Consequently, medication changes were delayed from hospital to home care. Participants said that the use of a uniform patient record system across healthcare organisations could prevent medication information flow challenges and incidents.

Not all places necessarily have the same patient record system as home care. If there are two or three [medication] changes in a week, it becomes an information flow problem. (*challenges and contributing factors*)—Interview 3

When clients go to the hospital, the patient record system views should be adapted so that the home care medication information is readily available in the hospital as well. This would ease to work on both parties, of course. (*prevention*)—Interview 7

Participants described that a few home care clients used health technology to take medication, such as a medicine‐dispensing robot. Hence, the healthcare professional could monitor the client's medication intake via a tablet computer. However, the client's medicine‐dispensing robot did not always work, resulting in the client not receiving their medication. This was challenging when the client had dementia and did not know how to inform the healthcare professionals, which participants saw as a contributing factor to challenges.

Then, of course, we have these medicine‐dispensing robots, but sometimes the robots do not work. Then, the client is left without medications, especially if the client has dementia and does not know how to contact home care, and usually, the alarm comes with a delay [if the medicine‐dispensing robot does not work]. (*challenges and contributing factors*)—Interview 3

### 5.5 Main Theme 3: The Healthcare Professionals' Individual Factors

*Challenges* were identified as incomplete actions by healthcare professionals and incorrect flow of medication information between them. *The contributing factors* were a lack of uniform practices among healthcare professionals, lack of resources, attitudes towards work, and individual characteristics. *Medication challenges and incidents can be prevented* by strengthening standard healthcare practices, improving individual factors of healthcare professionals, and increasing healthcare resources.

Participants described the healthcare professionals' incomplete actions that appeared between the home care system and other healthcare organisations providing medication care to clients and within the home care system. In these situations, work was not performed. As a result, the client's medication documents were not updated or checked, and the client's daily medication information was not documented. These challenges occurred when the hospital or home care physician forgot to update the medication list. Moreover, participants mentioned that there may also have been incorrect information in the medication documents, particularly when the client was discharged from the hospital.

The home care nurse did not document the client's medication information in the patient record system because the nurse did not consider the documentation to be necessary. The participants described that a few employees had outdated working practices; hence, the nurses wanted to document them on paper. Nurses only wanted to perform their client work. Consequently, the nurses were resistant to change and found it difficult to move away from these practices, even if new working methods contributed to client medication safety. Moreover, participants mentioned that old medication lists were observed in the client's home because home care professionals had forgotten to remove the old medication list, and the latest medication list was left in the home care professional's rucksack. These were factors contributing to the challenges. Consequently, participants suggested changing the nurses' attitudes, recognising their responsibilities, and taking an interest in improving clients' medication safety, which prevents the flow of medication information incidents. Participants also described the need for more training on medication information flow, considering the individual factors of home care professionals.

…often, medication lists are not up to date…you can find a surprising amount of information from them [medication list], and they would provide a lot of valuable information if they were up to date and clear. (*challenges*)—

Interview 6

It might be due to attitudes; not everyone finds paperwork enjoyable [which refers to documentation]. Some people think they just want to do client work. (*contributing factors*)—Interview 6

…everyone has the responsibility, in their own way, to manage their [medication] skills and maintain their skills. (*prevention*)—Interview 2

Participants mentioned that the absence of a registered nurse or physician contributed to clients' medication records not being up to date, or written work not being done. Participants felt that updating medication documents was mainly the responsibility of registered nurses and physicians. In particular, a registered nurse who was absent for an extended period or a shortage of nurses in the home care team created vulnerabilities. Participants said that improvements could be made as the number of home care professionals increases. Participants identified a long‐term lack of resources was a contributing factor that led to a lack of well‐being and overburden among home care professionals.

A lot of medication changes fall under the responsibility of the team registered nurse. However, if they are in the client's homes, they do not always have time to complete it [medication changes to the patient record system]. (*challenges and contributing factors*)—Interview 1

…if the resource situation is poor or there are no registered nurses, then the clients' medication lists are often left unupdated…. (*challenges and contributing factors*)—Interview 2

There are probably factors related to workplace well‐being behind this. We are overburdened, and the situation has lasted for a long time. (*contributing factors*)—Interview 6

Participants described that when a client was using a private health service, health centre, or hospital, the client's medication records were not updated when the client moved to home care. When a client was absent from home care services for an extended period and simultaneously visited several healthcare services within a short time, participants identified a challenge in that the client's current medication information did not transfer consistently from the beginning of the care period to discharge. Participants said that one contributing factor was the lack of uniform practices among healthcare professionals, whereby different organisations were unaware of each other's working practices.

The client first goes from us [home care] to the emergency unit, then from the emergency unit to another hospital, then from there to a rehabilitation hospital, and finally returns home from the rehabilitation hospital. They call us [home care] and say that medication changes have not been made, but in reality, changes to the medications have been made in another hospital or another place. (*challenges*)—Interview 1

Participants described that during client visits, permanent and substitute home care nurses did not always read the client's care plan; thus, care was provided from memory. This caused home care nurses to be unaware that the client's medication had changed. Participants said this as a lack of language skills and indifference to work on the part of home care professionals, which was seen as a contributing factor to challenges. Participants expressed particular concern about practical nurses who graduated during the COVID‐19 pandemic and had poor Finnish language skills due to remote teaching at that time. The participants described that recording, updating, and reviewing the client's medication prevented incidents of medication information flow. Writing a clear and concise care plan and reading the client's care plan could prevent medication‐related incidents. Moreover, the participants considered it important to strengthen the language skills of home care nurses.

…few people seem to read the care plan…. (*challenges*)—Interview 5

Language skills might be one reason why instructions, care plans, and medication lists are not read correctly, leading to these incidents. (*contributing factors*)—Interview 4

You have to be prepared for changes [regarding medication]; they can happen anytime, so you can't trust that if you visited in the morning and go again in the daytime, nothing has changed. (*prevention*)—Interview 7

### 5.6 Main Theme 4: Client‐Related Challenges

*The challenges* were related to situations where the client independently visited healthcare services, did not follow the healthcare professionals' instructions on taking medication, or lacked medication information. Client‐related factors were *contributing factors*. Guiding clients *can prevent medication‐related challenges and incidents*.

Participants described situations as challenging where home care clients independently dealt with different healthcare organisations. The client provided healthcare professionals with insufficient information about the medication. In addition, after returning home, the client did not inform home care about medication changes. In this case, home care professionals did not receive the client's medication change information, even though they managed the client's medication.

I have experienced a small challenge when the home care nurses manage the client's medication matter, but for some reason, the clients or their relatives deal with health centres, hospitals, or other services. Consequently, medication changes are not communicated to home care. (*challenges*)—Interview 7

Participants described that clients sometimes took medication themselves from the pharmacy, resulting in them receiving extra medication or not taking the medication as required. Consequently, the client did not follow the healthcare professionals' instructions regarding taking the medication. In these cases, the contributing factor was the client's illness, such as dementia or substance abuse. The complexity increased when the client did not have a medication list at home, even though they dispensed the medications themselves. Participants said increasing client guidance about medications and awareness of their medication could prevent these incidents.

…for example, a client with dementia goes to the pharmacy to get pills or sends relatives to get them. Suddenly, all the pills are mixed up because they are retrieved from two different pharmacies, leading to confusion the client's medication care. (*challenges and contributing factors*)—Interview 2

Moreover, that clients are aware of their own well‐being [referring to medication]. (*prevention*)—Interview 2

## 6 Discussion

Our study described the challenges related to the flow of medication information in home care, their contributing factors, and home care registered nurses' and nurse leaders' views on preventing them. This study confirmed existing knowledge, as the information regarding the flow of medication information is insufficient.

*The complexity of home care work in cooperation and the medication process* is seen as a challenge for medication information flow. It is complex because the home care medication process includes several stages and involves various healthcare professionals, the pharmacy, the client, and the client's relatives. The complexity is increased because the client may take medications in several places, such as a dose box, a dose delivery bag, and a medicine‐dispensing robot. Additionally, the client's medication plan may change rapidly. This complexity shows that healthcare professionals, clients, and relatives require uniform medication information flow practices. Berland and Bentsen (2017) reported that healthcare professionals continually strive to create new routines with leadership to improve safer medication distribution. Our study confirmed the existing knowledge that pharmacists, as part of the home care team, prevented incidents of medication information flow, as reported by Meyer‐Massetti, Meier, and Guglielmo (2018). Kee et al. (2018) reported that using pharmacists to ensure the accuracy of medication lists when a client moved from a hospital to primary care prevented medication incidents.

Challenges to cooperation manifested themselves differently in this study; these were internal to home care, between home care and other healthcare organisations, and between home care and clients and relatives. This is consistent with the communication structure framework by Kuziemsky et al. (2009), which is divided into internal and external team communication processes. In this study, not all healthcare professionals received the client's medication information, or these were delayed from the hospital or the health centre. Berland and Bentsen (2017) and Vellonen et al. (2023) also identified a lack of information sharing between physicians and home care nurses. We also found that medication information flowed informally within the home care team, consistent with previous research. Vellonen et al. (2023) identified this challenge in their study when the client's information was passed through a crumpled note in the home care team. Overall, cooperation challenges and fragmented healthcare systems increased medication incidents, particularly during client transitions from hospital to home care. Squires et al. (2020) also sought to understand the medication management challenges from the perspective of home care professionals. They observed that fragmented client care and the distribution of care among different healthcare professionals increased the incidents and challenges of taking medications as directed. In a review by Dionisi et al. (2022), medication incidents occurred mainly during the client transition phase, where inaccuracies were observed in transition documentation, such as medication lists.

*Technology‐related challenges* were identified in this study, such as weaknesses in the functionality of the home care patient record system, fragmented medication information in the patient record system, and the lack of a uniform patient record system between healthcare professionals. Lindblad et al. (2017) reported that the electronic medical record is perceived as complex, involving the simultaneous use of several pages to write a prescription. A study by Vellonen et al. (2023) also observed the lack of a uniform patient record system. These results suggest that more attention should be paid to communication between different healthcare organisations. Thus, patient record systems are not uniform across healthcare organisations. Participants suggested simplifying the patient record system in this study to avoid incidents involving medication information flow. Simplifying a patient record system is challenging. Therefore, attention should be paid to improvements, such as medication and patient record system training for nurses. Previous studies also confirmed that regular training on medication management increases client safety (Khalil and Lee 2018; Strube‐Lahmann et al. 2022).

*The healthcare professionals' individual factors* were identified as a challenge to medication information flow. Incomplete actions by healthcare professionals and incorrect medication information flow between healthcare professionals are challenges in home care. The factors contributing to this were healthcare professionals' attitudes towards work, individual characteristics such as the negative attitudes of home care professionals towards work tasks, and human factors. A study by Vellonen et al. (2023) identified incomplete actions and incorrect medication communication as factors contributing to medication incidents. Incomplete actions occurred in situations where a task was not completed; for example, when no nurse added a client's visit to the patient record system, resulting in no visit and medication not being administered (Vellonen et al. 2023). In a study by Säfholm et al. (2019), physicians did not update 84% of the client's medication lists in the primary healthcare setting.

Additionally, participants in this study felt that incomplete actions were due to indifference and that the work was not considered important, such as not documenting medication information because the nurses wanted to focus only on client care at home. Although participants identified that the information flow improved when they documented medication information in the patient record system. These negative attitudes of home care professionals towards their work tasks provide new insights into the factors contributing to the flow of medication information incidents. Participants also described busyness, insufficient healthcare professionals, and overburden as factors contributing to challenges. Härkänen et al. (2020) also discovered that limited healthcare resources can contribute to medication incidents.

In this study, participants described that medication documents may have been incorrect during the transition phase, particularly when the client was discharged from the hospital. Berland and Bentsen (2017) reported that when a client is discharged from the hospital, the client's medication is unclear, or discharge instructions are incorrect. Nurses often had to call the hospital again to ask for clarification on unclear medication. Registered nurses expressed concern that contacting physicians about a client's medication would be difficult. This challenge was compounded by physicians not returning calls to registered nurses (Berland and Bentsen 2017).

This study identified *client‐related challenges*, which expanded previous knowledge. Clients did not always inform home care professionals of medication changes if they visited another healthcare service. Previous research has identified a lack of information sharing from clients to home care (Vellonen et al. 2023). The client should inform the home care team about medication changes. However, this is challenged when the client has dementia and can pick up their medication from the pharmacy.

### 6.1 Strengths and Limitations of the Work

This study provided a broad perspective on the challenges of medication information flow. All home care nurses and nurse leaders in a city had the opportunity to participate in the study. The participants were registered nurses and home care leaders. It would have been beneficial to interview practical nurses, as they work closely with clients and cooperate with their relatives and the multidisciplinary healthcare team.

A limitation of this study is that the sample only focused on a home care organisation in one city. Nevertheless, the participant number was sufficient because the data was saturated (Polit and Beck 2021). The perspectives of clients and their relatives would have further enriched the findings. The interviews were conducted remotely since the participants were far from each other. However, it may be possible that face‐to‐face interviews would have produced a more in‐depth discussion. On the other hand, studies have not found differences between face‐to‐face and remote interviews regarding information richness (Jones et al. 2022).

### 6.2 Recommendations for Further Research

In the future, it would be useful to investigate medication information flow incidents from the perspective of hospital staff, health centres, and pharmacies to gain a deeper understanding of medication information flow in home care and cooperation between different healthcare providers. For example, the challenges should be clarified in the medication information flow when the client is discharged. In addition, the perspective of the client and relatives and how they perceive cooperation with healthcare providers on the flow of medication information should be studied.

Previous studies have identified medication‐related incidents, the factors that contribute to them, and ways to prevent medication incidents. Therefore, it would be useful to implement interventions to improve medication information flow and evaluate their effectiveness. The studies could provide information on effective home care practices.

### 6.3 Implications for Policy and Practice

This study recommends:

- During care transitions, healthcare professionals should agree on standard healthcare practices regarding the flow of medication information.
- It is recommended that communication tools, such as patient record systems, lean boards, and checklists, be utilised in home care to facilitate an efficient flow of information among care providers.
- Increasing the number of home care professionals.
- Pharmacists are an important part of the home care team.

## 7 Conclusion

The home care environment is complex from the perspective of medication information flow. More resources should be allocated to home care. It is essential to ensure that there are enough practical nurses and registered nurses and that nurses have time to reflect on the medication information flow practices in the home care team and, more broadly, with other healthcare providers.

## Author Contributions

M.V., M.H. and T.V.: Made substantial contributions to conception and design, or acquisition of data, or analysis and interpretation of data. Involved in drafting the manuscript or revising it critically for important intellectual content. Given final approval of the version to be published. Each author should have participated sufficiently in the work to take public responsibility for appropriate portions of the content. Agreed to be accountable for all aspects of the work in ensuring that questions related to the accuracy or integrity of any part of the work are appropriately investigated and resolved.

## Conflicts of Interest

The authors declare no conflicts of interest.

## Supporting information


Data S1.

## Data Availability

Research data are not shared.
